# Central line associated blood stream infectious cause by multi drug resistance coagulaso-negative Staphylococci in newborns from neonatial intensive care units in Poland

**DOI:** 10.1186/1753-6561-5-S6-P205

**Published:** 2011-06-29

**Authors:** M Brzychczy-Wloch, J Wojkowska-Mach, M Borszewska-Kornacka, M Sulik, E Gulczynska, P Heczko

**Affiliations:** 1 Chair of Microbiology, Jagiellonian University Medical College, Krakow, Poland; 2 Neonatal and Intensive Care Department , Medical University of Warsaw , Poland; 3 Duchess Anna Mazowiecka, Teaching Hospital, Warsaw, Poland; 4 Polish Mother`s Memorial Hospital, Lodz, Poland

## Introduction / objectives

The project aims to analyze epidemiology and microbiology of Central Line Associated Blood Stream Infections (CLABSI) cause by Multi Drug Resistance Coagulaso-Negative Staphyloccoci (MDRCoNS) in children with very low birth weight (VLBW).

## Methods

Data collection on CLABSI in VLBW newborns was made prospectively for 2009 year. Study covered 386 neonates of birth weight <1500g in 2 Polish NICUs (A and B), among which 55 cases of CLABSI were detected. CoNS strains isolated from 26 newborns with CLABSI, were determined in blood in the automatic system Vitek, drug resistance was determined by disc diffusion method.This study was supported by a grant no. NN401615340.

## Results

Birth-weight and gestational age were significantly different between newborns in NICU-A and B (*P*<0.01). The CLABSI incidence per 1000 CVC/pds (patient days) in NICU-A and B were 8.5 and 5.2, respectively (RR1.6). CVC utilization in NICU-A and NICU-B were 0.5 and 0.4, respectively. The most common etiological factors of CLABSI were CoNS (66%). The dominant species were *S.epidermidis* (63%), *S.haemolityicus* (20%), *S.warneri* (8%), *S.hominis* (5%), *S.xylosus* (2%) and *S.capitis* (2%). Among 26 newborns with CoNS BSI, 2 children had polymicrobial infections caused by *S. haemolyticus* and *S. epidermidis.* Resistance to methicillin, macrolides, aminoglycosides and fluoroquinolones was detected in 98%, 70%, 78% and 43% of isolates, respectively. All methicillin resistant CoNS strains had *mec*A gen.

## Conclusion

Understanding the epidemiology of CLABSI in VLBW neonates is a key step in development of targeted prevention strategies and reduce antibiotic consumption.

## Disclosure of interest

None declared.

